# Predicting 30-Days Hospital Readmission for Patients with Heart Failure Using Electronic Health Record Embeddings: Comparative Evaluation

**DOI:** 10.2196/73020

**Published:** 2025-11-25

**Authors:** Prabin Shakya, Ayush Khaneja, Kavishwar B Wagholikar

**Affiliations:** 1 Laboratory of Computer Science, Massachusetts General Hospital, 399 Revolution Drive, 7th Floor, Boston, MA, 02145, United States, 1 8595360114; 2 Harvard Medical School, Boston, MA, United States

**Keywords:** heart failure, machine learning, embedding techniques, electronic health record, EHR, hospital readmission

## Abstract

**Background:**

Heart failure (HF) is a public health concern with a wider impact on quality of life and cost of care. One of the major challenges in HF is the higher rate of unplanned readmissions and suboptimal performance of models to predict the readmissions. Hence, in this study, we implemented embeddings-based approaches to generate features for improving model performance.

**Objective:**

The objective of this study was to evaluate and compare the effectiveness of different feature embedding approaches for improving the prediction of unplanned readmissions in patients with heart failure.

**Methods:**

We compared three embedding approaches including *word2vec* on terminology codes and concept unique identifier (CUIs) and BERT on descriptive text of concept with baseline (one hot-encoding). We compared area under the receiver operating characteristic (AUROC) and *F*_1_-scores for the logistic regression, eXtream gradient-boosting (XGBoost) and artificial neural network (ANN) models using these embedding approaches. The model was tested on the heart failure cohort (N=21,031) identified using least restrictive phenotyping methods from MIMIC-IV dataset.

**Results:**

We found that the embedding approaches significantly improved the performance of the prediction models. The XGBoost performed better for all approaches. The *word2vec* embeddings (0.65) trained on the dataset outperformed embeddings from pre-trained BERT model (0.59) using descriptive text.

**Conclusions:**

Embedding methods, particularly *word2vec* trained on electronic health record data, can better discriminate HF readmission cases compared to both one-hot encoding and pre-trained BERT embeddings on concept descriptions making it a viable approach of automation feature selection. The observed AUROC improvement (0.65 vs 0.54) may support more effective risk stratification and targeted clinical interventions.

## Introduction

Heart Failure (HF) is a major and growing public health concern, affecting around 3% of the adult population of high-income countries, with increasing prevalence in low and middle-income countries [1,2]. It is estimated that 56.2 million people worldwide are affected by HF [2]. HF is also associated with high rates of unplanned readmissions, which strain the health care system and diminish patient quality of life. The 1-year readmission rate is as high as 53% globally and 59% in the United States [3]. Given the significant burden imposed by readmissions, healthcare providers strive to identify patients with HF at risk for readmission. Several predictive models have been developed for this purpose, including traditional statistical models and machine learning models. However, their predictive performance remains suboptimal in real-world applications, with area under the receiver operating characteristic curve (AUROC) of 0.6 when using electronic health record (EHR) data [4].

Medical codes such as International Classification of Diseases (ICD) codes, procedure codes, and medication identifiers vary in structure and granularity, introducing complexity and high dimensionality into the data. These high-dimensional spaces make it difficult to extract meaningful patterns for prediction. Natural language processing and embedding techniques such as word2vec and BERT offer a promising approach to address these challenges by transforming discrete medical codes into continuous vector representations. These embeddings capture semantic relationships between medical codes, enabling models to understand the similarities and differences between related diagnoses and procedures [5,6]. Several studies have demonstrated that embedding methods may improve the performance of predictive models in healthcare by reducing dimensionality while preserving relevant clinical information [6,7].

To address these challenges, our study, investigates the utility of EHR embedding techniques and their performance in the prediction of 30-day hospital readmissions among patients with HF, leveraging the open data available in MIMIC-IV dataset. The 30-day readmission metric is widely used in healthcare policy and quality assessment. In the United States, the Center of Medicare and Medicaid Services includes this metric in the Hospital Readmissions Reduction Program, which penalizes hospital excess readmissions [8]. As such, accurate prediction of 30-day readmissions risk has direct implications for hospital reimbursement and patient care planning.

We compared two embedding approaches: (1) *word2vec* embeddings trained on terminology codes and Unified Medical Language System (UMLS) Concept Unique Identifier (CUIs) and (2) BERT embeddings derived from concept descriptors. We hypothesize that embeddings derived from structured medical codes capture patient-level clinical patterns more effectively than embeddings generated from descriptive text. This is because code-based embeddings reflect co-occurrence and usage patterns in real clinical settings, while text-based embeddings may lack the specificity and contextual relevance needed for structured prediction tasks.

Word2vec is a widely used embedding technique used to represent words (or codes) in a vector space, trained using shallow neural networks on text corpora [9], In the clinical domain, word2vec has been successfully used to embed medical concepts from EHR data [10-12]. In contrast, BERT is a transformer-based language model that generates contextualized embeddings by capturing the position and surrounding context of each word [13]. BERT and its biomedical adaptations such as BioBERT and BioClinicalBERT have shown promise for a range of tasks in biomedical and clinical domains [14-17]. Among these, BioBERT is pretrained on PubMed abstracts, while BioClinicalBERT extends it with clinical narratives from MIMIC. Lee et al [6] demonstrated that textual descriptions of ICD codes can be effectively used for semantic-preserving vector representations.

While BERT provides contextualized embeddings that excel in natural language understanding, its effectiveness in representing brief and structured medical concept descriptions remain uncertain. In contrast, word2vec, trained on real-world sequences of medical codes, may better capture clinically relevant co-occurrence patterns. In this study, we aim to empirically evaluate whether code-based embeddings (eg, word2vec on medical codes) outperform text-based embeddings (eg, BERT on concept descriptions) in predicting 30-day hospital readmissions, given the structured nature of EHR data.

## Methods

### Study design

For this study, we used Medical Information Mart for Intensive Care (MIMIC)-IV version 2.2, a publicly available dataset released in January 2023. The MIMIC-IV dataset contains comprehensive, de-identified EHR data for patients admitted to the Beth Israel Deaconess Medical Center between 2008 and 2019. The dataset includes 299,712 patients and 431,231 hospital admissions [18]. Diagnoses are coded using the ICD, with both 9th and 10th Clinical Modification (CM) revisions. Procedures are recorded using both *ICD*-9 and *ICD*-10 Procedure Coding System codes. Medications are recorded with the National Drug Code (NDC) along with the Drug name, route, strength, etc. To develop predictive models for HF readmission, we used data from the hospital module capturing demographic information, diagnosis, medication and procedure codes.

We identified patients with HF using the least restrictive phenotyping method, which defines HF-positive cases as any patient with at least one relevant *ICD*-9 or *ICD*-10 code for HF [19]. We included HF-positive patients only for this study. 30-day readmission was defined as any subsequent admission occurring within 30 days of discharge, regardless of the reason for readmission. Patients were excluded from the cohort if they died (n=1475), or the length of stay was less than one day (n=1740) at the indexed admission. The exclusion criteria were applied because patients who died during the index admission were not at risk for readmission, and the definition of ‘inpatient’ becomes ambiguous for patients with hospital stay of less than one day. Admission occurring after the index admission were also excluded, as our objective was to predict readmission following the index HF hospitalization.

**Figure 1.**
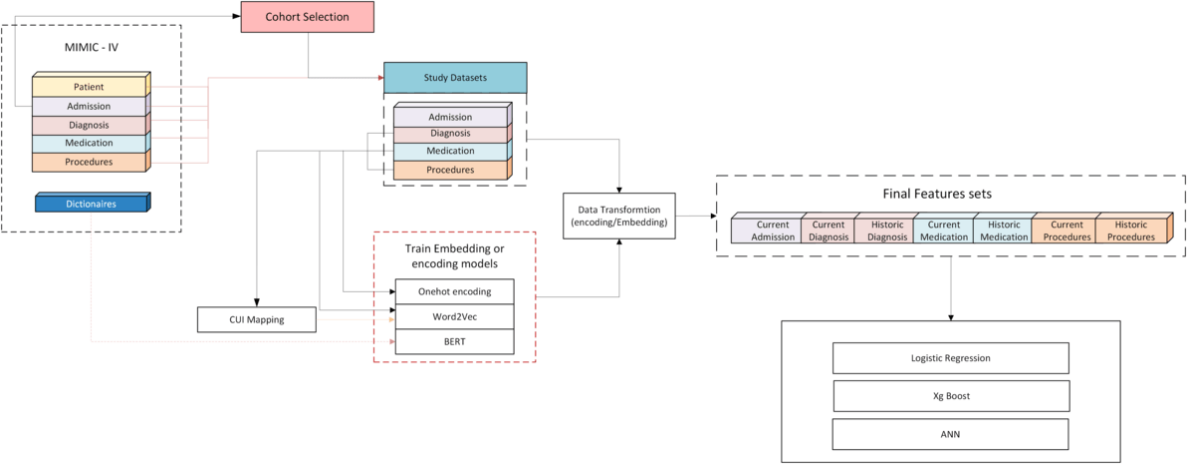
Graphical summary of the Study. CUI: Concept Unique Identifiers ; MIMIC-IV: Medical Information Mart for Intensive Care - IV (Database).

Data preprocessing was structured into three main components: admissions, clinical data extraction, and embedding preparation:

1. Admissions: Admissions were categorized as indexed, historic, and future admissions. Indexed admissions were defined as the first hospitalization with a diagnosis of HF. All the admissions prior to indexed admissions were classified historic admission and any subsequent hospitalizations as future admissions. We only developed models for predicting readmission after the index admissions. We included age at admission, length of stay and gender from indexed admissions as features.
2. Clinical Data extraction: We extracted diagnoses, procedures, and medications from structured data for selected cohorts. These were split into current if date of record corresponds to the index admission and historic if it precedes the indexed admissions. We applied different encoding or embedding methods to these extracted data. We applied one-hot encoding for baseline. The embeddings approaches applied are described below. The current and historic data are combined with indexed admission features to create the final features sets (Figure 1).
3. Embedding: We applied three embedding approaches to the feature matrix to capture the semantic representations in the data, to output a new feature matrix.
  - Word2Vec on Terminology Codes: ICD codes were concatenated with their version (ICD-9 or ICD-10) to form unique identifiers that account for overlapping codes between the two versions. For medications, we used NDC codes, excluding drugs that lacked NDC identifiers. Each unique code (diagnosis, procedure, or medication) was treated as a word, and the list of codes for each patient encounter was considered as a document. The word2vec model was trained using the skip-gram with negative sampling (SGNS) algorithm. The subset of training dataset was used to finetune parameters for word2vec (please refer to Table S1 in Multimedia Appendix 1 and Figure 1). Based on the finetuning experiments, we selected the context window size of 10, resulting in 200-dimensional embedding vectors. Separate *word2Vec* models were trained for diagnoses, procedures, and medications.
  - Word2Vec on UMLS CUIs: To map ICD and procedure codes to UMLS CUIs, we implemented a hybrid code and string-matching approach. First, codes were matched directly to UMLS using a code-matching algorithm. For unmapped code, we truncated the first three characters and attempted matching again. If multiple CUIs were returned, we selected the most generic code. For Medications, NDC codes were matched and for unmapped codes, we used a string search from the drug name. These mapped CUIs were then treated as ‘word’ and trained in the same manner as recorded medical codes using Word2Vec.
  - BERT on Descriptive Text: For each medical code, we utilized its long-form descriptor to generate embeddings using pre-trained BERT models. We used BioClinicalBERT, which is specialized for clinical texts, to generate context-aware embeddings from the long descriptions provided in the MIMIC-IV datasets. For medication, as long descriptions were not available, we created one by concatenating multiple columns (drug, prod_strength, dose_val_rx, dose_unit_rx, form_val_disp, form_unit_disp and route) from the prescriptions table for example - “Docusate Sodium 100 mg Tablet 100 mg 1 Tab 2.0 Oral.” This approach captures semantic meaning and context-specific nuances that are often lost when only the codes are used.

We developed multiple machine-learning models to predict 30-day readmissions. The embeddings generated from each approach were used as input features for these models. We trained and evaluated three machine learning algorithm—logistic regression, and eXtream gradient-boosting (XGBoost) and artificial neural network (ANN). A feedforward ANN consisting of three fully connected layers with Tanh activation and dropout for regularization with progressively reduced dimensionality and outputs a probability via a final sigmoid activation was implemented. Like *word2vec* parameters finetuning, we run multiple experiments with a combination of different parameters and oversampling of minor classes using Synthetic Minority Oversampling Technique (SMOTE). Model performance was assessed using standard metrics such as Area under the receiver operating characteristic curve (AUROC), and *F*_*1*_*-*score. (please refer to supplement file in Multimedia Appendix 1 for the link to the source code).

### Ethical Considerations

The study used the MIMIC dataset, which contains deidentified health-related data and is publicly available through PhysioNet platform. Access to the dataset requires completion of the “Data or Specimen Only Research” course and acceptance of the data use agreement. The institutional review board at Mass General Brigham determined that the project does not meet the criteria for human subject research, as the dataset used does not contain any identifiable patient information. Since this study used an open dataset available in the public domain that was published by another research group, informed consent and opt-out procedures were managed by the original investigators. Therefore, no direct consent or interaction with participants was required for this study.

## Results

**Figure 2.**
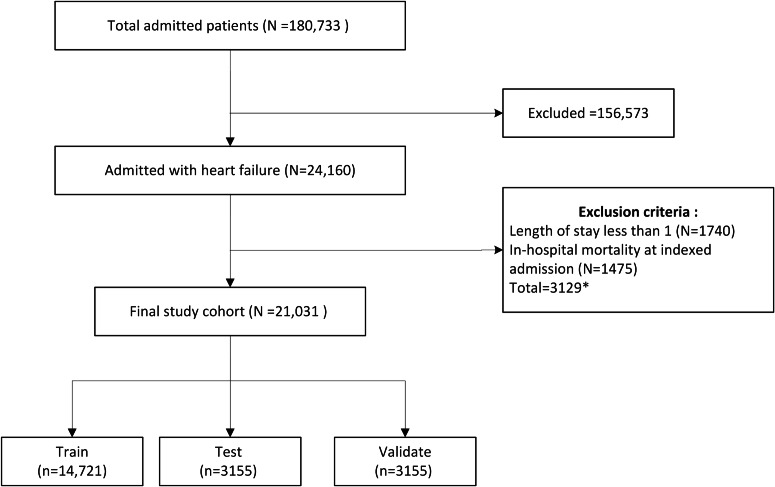
Flow diagram study cohort selection. *The total number of unique excluded patients accounts for 86 individuals who met both exclusion criteria.

A total of 21,031 patients were included in the study with 3933 (19%) experiencing a 30-day readmission. This cohort was randomly split into training with 14721 patients (70%), test with 3155 patients (15%), and validation with 3155 patients (15%) sets (Figure 2). The average age at indexed admission was 73 years, the mean number of admissions was 2, the mean number of ICD codes used was 25 and the mean length of stay is 8 days. The number of admissions (*P*<.001, *t*-statistic=10.83), number of ICD codes (*P*<.001, t-statistic=12.71), and length of stay (*P*<.001, *t*-statistic=10.41) are higher among patients with 30-day readmission, and there is no significant difference in age at index admission (*P*=.40, *t*-statistic=−0.851) and the number of male versus female (*P*=.63, odds ratio =1.017). This is true in all splits of datasets as well (Table 1). The rate of 30-day readmission was 19% in the Cohort, 19% in the Train dataset, 17% in the test set, and 19% in the validate set.

**Table 1.** Cohort characteristics (N=21,031).

Variables	Total	Negative	Positive	*P* value
Sex, n				.63^a^
Female	9971	8120	1851	
Male	11,060	8978	2082	
Admissions, mean, (SD)	2.07 (2.88)	1.98 (2.44)	2.52 (4.28)	<.001^b^
ICD codes, mean (SD)	24.59 (18.12)	23.83 (16.93)	27.89 (22.25)	.001^b^
Length of stay, mean (SD)	7.83 (8.57)	7.53 (8.04)	9.11 (10.48)	.001^b^
Age at index admission, mean (SD)	73.36 (13.55)	73.39 (13.55)	73.2 (13.56)	.40^b^
Total	21,031	17,098	3933	—^c^

a *χ*2 test

b *t* test

c Not applicable.

We used one-hot encoding as baselines, which yielded AUROC scores of 0.54 and *F*_1_-scores ranging from 0.24 to 0.26 across validation and test sets. The embedding-based approaches consistently outperformed the baseline across all models. Logistic regression, XGBoost, and ANN models achieved AUROCs ranging from 0.59 to 0.64, 0.59 to 0.65 and 0.59 to 0.64, respectively. Among the embedding strategies, BERT embeddings from descriptive text performed worst, while the best AUROC (0.65) was achieved using *word2vec* on CUIs with XGBoost. F1 scores varied modestly, ranging from 0.32‐0.37 (validation) and 0.29‐0.34(test) (Table 2). The best logistic regression results were obtained using L2 regularization with C of 0.01 for both *word2vec* and BERT embeddings. For XGBoost, optimal performance was achieved with max depth of 3 and learning rate of 0.01. For ANN models, a learning rate of 0.001, dropout rate of 0.3, and hidden size of 384 yield the best outcomes across most experiments. Logistic regression and XGBoost performed better without oversampling, whereas the ANN showed improved performance when training set was oversampled to address class imbalance (please refer to Table S2 in Multimedia Appendix 1).

**Table 2.** Best results of different experiments by model.

Experiments and models	AUROC^a^ validation	*F*_1_ validation	AUROC test	*F*_1_ test
Baseline				
Logistic regression	0.54	0.26	0.54	0.25
XGBoost^b^	0.54	0.25	0.54	0.25
ANN^c^^,^^d^	0.54	0.24	0.54	0.24
Word2vec on terminology code				
Logistic regression	0.64	0.37	0.64	0.34
XGBoost	0.65^e^	0.38^e^	0.65^e^	0.33
ANN	0.64	0.37	0.64	0.34
Word2vec on CUIs^f^				
Logistic regression	0.63	0.35	0.62	0.32
XGBoost	0.63	0.36	0.65^e^	0.35^e^
ANN	0.60	0.34	0.61	0.32
BERT on descriptive text				
Logistic regression	0.59	0.32	0.59	0.30
XGBoost	0.59	0.32	0.59	0.29
ANN	0.59	0.33	0.59	0.31

a AUROC: area under the receiver operating characteristic curve.

b XGBoost: eXtreamextreme gradient-boosting.

c Oversampling of training dataset using synthetic minority oversampling technique.

d ANN: artificial neural network.

e best score.

f CUI: Concept Unique Identifier.

## Discussion

### Principal Findings

The study presents the systematic comparison of embedding techniques for predicting 30-day heart failure readmission using EHR data. Our analysis of 21,031 heart failure admission from MIMIC-IV dataset demonstrates that the use of *word2vec* embedding trained on the patient data achieved superior performance compared to traditional one-hot-encoding and pretrained language model (BERT).

The *word2vec* on CUIs with XGBoost had AUROC of 0.65, which is substantial improvement of 0.11 over the one-hot-encoding baseline, and modest improvement of 0.06 over pre-trained BioClincial_BERT model. The corresponding F_1_-score improvement of 0.10 and 0.04, respectively demonstrates enhanced performance of the word2vec approach.

**Figure 3.**
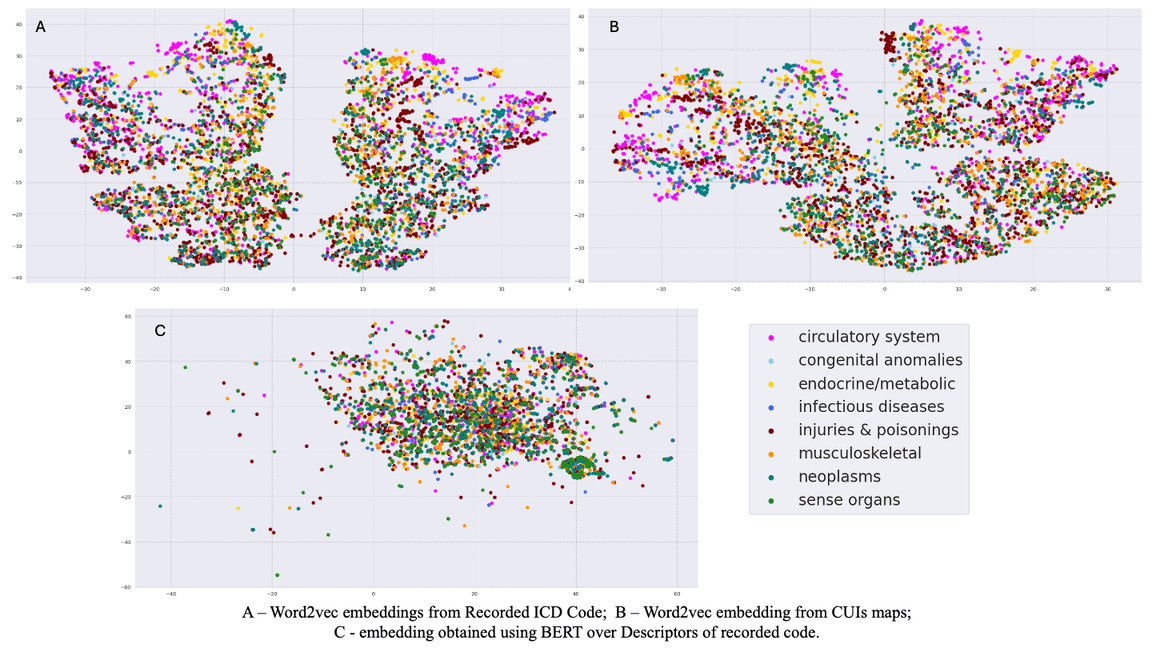
T – SNE plot of diagnosis code embeddings with encoded categories.

We investigated the performance gains for word-2-vec using dimensionality reduction analysis. A t-distributed stochastic neighbor embedding (Figure 3) revealed that word2vec embeddings facilitated more coherent clustering of diagnostically related codes, implying superior capture of clinically relevant semantic relationships.

Overall our findings align with literature in data-driven approaches for readmission prediction [3,7,20,21], while revealing important improvements that can be obtained by using embeddings. However our results were inferior compared to previous studies that utilized expert-crafted features, where the AUROC scores exceeding 0.8 [22,23]. For instance, Pishgar et al [22] reported AUROC of 0.70 using features selected as the event logs with ANNs, and Ben-Assuli et al [23] achieved 0.88 using mix of ML algorithm and expert-selected features with XGBoost. This performance differential highlights the trade-off between automated feature engineering and use of human-experts for feature selection. This is consistent with findings across other clinical areas, where domain expertise has been shown to significantly enhance predictive performance.

Notably, our results suggest that dataset-specific word2vec embeddings may outperform sophisticated pre-trained language models. This is likely because approaches that directly model co-occurrence patterns within the target dataset may capture more task-specific signal as compared to models trained on broader textual corpora.

Overall our study fills a significant gap in the literature by offering a systematic evaluation of embeddings strategies, thereby providing evidence-based guidance for selecting the appropriate embedding method for clinical prediction tasks.

The study has several limitations. Patient encounters were modeled as static collection of medical codes without considering temporal patterns. The study did not evaluate the portability of the models as the dataset is limited to a single institution. Our evaluation focused on traditional machine learning approaches (logistic regression, XGBoost, and ANN), which may not fully exploit the representational capacity of embedding methods. Future research should explore temporal sequence modeling with transformer-based architectures, graph-based representation learning, and multimodal data to capture richer clinical context. Validation across multiple institutions and diverse population is essential to demonstrate portability.

### Conclusion

In conclusion, this study demonstrated that embedding techniques, particularly word2vec trained on study dataset is a viable automated approach for heart failure readmission prediction. Further studies are required to investigate if task-specific representation learning may be more effective than use of general-purpose models for specific clinical applications.

## Supplementary material

10.2196/73020 Multimedia Appendix 1 Fine-tuning the *word2vec* model.

## Abbreviations

ANN: Artificial neural network
AUROC: area under the receiver operating characteristic curve
CUI: Concept Unique Identifier
EHR: electronic health record
HF: heart failure
ICD: International Classification of Diseases
MIMIC: Medical Information Mart for Intensive Care
NDC: National Drug Code
UMLS: Unified Medical Language System
XGBoost: eXtream gradient-boosting
