# Supplementary material for: Predicting 30-Days Hospital Readmission for Patients with Heart Failure Using Electronic Health Record Embeddings: Comparative Evaluation
Source: JMIR Med Inform. 2025 Nov 25;13:e73020. doi: 10.2196/73020 (PMC12646029; doi:10.2196/73020)
Supplement: Multimedia Appendix 1 [file medinform-v13-e73020-s001.docx]

# Supplementary File

## Fine Tuning the word2vec model.

The word2vec model was finetuned using the training dataset. The performance of logistic regression was compared across different vector sizes (100,200,300,400,500) and window sizes (2, 5, and 10). The AUC, F1-Score, Recall and Precision were highest for the vector size 200 and window size of 10; these parameters were subsequently used for the main experiment.

Table 1 : Results of word2vec Fine-tuning

| **Vector size** | **window** | **AUC** | **precision** | **recall** | **f1** |
| --- | --- | --- | --- | --- | --- |
| 100 | 2 | 0.64 | 0.30 | 0.57 | 0.39 |
| 100 | 5 | 0.64 | 0.30 | 0.58 | 0.39 |
| 100 | 10 | 0.65 | 0.31 | 0.60 | 0.41 |
| 200 | 2 | 0.64 | 0.30 | 0.57 | 0.39 |
| 200 | 5 | 0.65 | 0.32 | 0.61 | 0.42 |
| **200** | **10** | **0.66** | **0.32** | **0.62** | **0.42** |
| 300 | 2 | 0.64 | 0.30 | 0.58 | 0.39 |
| 300 | 5 | 0.65 | 0.31 | 0.60 | 0.41 |
| 300 | 10 | 0.66 | 0.31 | 0.60 | 0.41 |
| 400 | 2 | 0.64 | 0.30 | 0.58 | 0.39 |
| 400 | 5 | 0.65 | 0.31 | 0.60 | 0.41 |
| 400 | 10 | 0.65 | 0.31 | 0.60 | 0.41 |
| 500 | 2 | 0.64 | 0.30 | 0.58 | 0.39 |
| 500 | 5 | 0.65 | 0.31 | 0.60 | 0.41 |
| 500 | 10 | 0.66 | 0.31 | 0.60 | 0.41 |


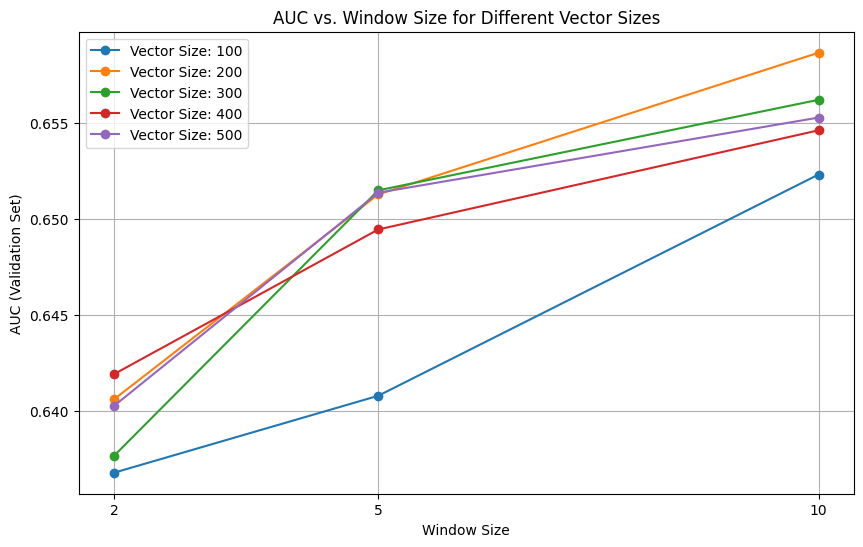


Fig 1 : AUROC Scores by window size and vector size using logistic regression model.

Model Parameter Testing
We assessed the impact of SMOTE-based oversampling and hyperparameter tuning across three models: logistic regression, XGBoost, and artificial neural networks (ANN). For logistic regression, we experimented with combinations of L1 and L2 penalties and regularization strengths (C values). For XGBoost, we tuned tree depth and learning rate, while for ANN, we varied the dropout rate, hidden layer size, learning rate, and batch size. Our results showed that oversampling had minimal impact on the performance of logistic regression and XGBoost. However, ANN performance improved significantly with oversampling, with F1 scores increasing from 0.24 to a maximum of 0.37.

In the baseline setting, the best ANN configuration (learning rate = 0.001, dropout = 0.2, hidden size = 64, epochs = 50, batch size = 32) with SMOTE achieved an AUROC of 0.54 and F1 score of 0.24. Logistic regression with L1 regularization (C = 0.01) and XGBoost with max depth = 2 and learning rate = 0.01, both without oversampling, achieved AUROC of 0.54 and F1 score of 0.26.

In Experiment 1 using original terminology codes, the best results were achieved by ANN with SMOTE (AUROC = 0.64, F1 = 0.37), logistic regression with L2 regularization (C = 0.01, AUROC = 0.64, F1 = 0.37), and XGBoost (depth = 3, learning rate = 0.01) with AUROC = 0.65 and F1 = 0.38.

In Experiment 2 using mapped CUIs, ANN again benefited from SMOTE (AUROC = 0.60, F1 = 0.34), while logistic regression (L1, C = 0.1) and XGBoost (depth = 3, learning rate = 0.01), both without oversampling, performed comparably (AUROC = 0.63, F1 = 0.35–0.36).

In Experiment 3, which used pre-trained BERT-based embeddings, the best ANN configuration with SMOTE achieved AUROC = 0.59 and F1 = 0.33; logistic regression (L2, C = 0.01) without oversampling matched this performance, and XGBoost (depth = 2, learning rate = 0.01) yielded slightly lower scores (AUROC = 0.58, F1 = 0.32). Overall, while traditional models were robust enough to oversample, ANN models showed marked improvements with SMOTE, particularly in settings with richer feature representations.

Table 2 : Model performance with varied parameters and oversampling approach.

| experiment | model | smote | params | Validate | | Test | |
| --- | --- | --- | --- | --- | --- | --- | --- |
|  |  |  |  | AUC | F1 | AUC | F1 |
| baseline | ANN | FALSE | {'learning_rate': '0.001', 'dropout_rate': '0.3', 'hidden_size': '384', 'epochs': '50', 'batch_size': '32'} | 0.53 | 0.03 | 0.54 | 0.02 |
| baseline | ANN | FALSE | {'learning_rate': '0.001', 'dropout_rate': '0.3', 'hidden_size': '128', 'epochs': '50', 'batch_size': '32'} | 0.54 | 0 | 0.55 | 0 |
| baseline | ANN | FALSE | {'learning_rate': '0.001', 'dropout_rate': '0.2', 'hidden_size': '64', 'epochs': '50', 'batch_size': '32'} | 0.55 | 0 | 0.55 | 0 |
| baseline | ANN | TRUE | {'learning_rate': '0.001', 'dropout_rate': '0.3', 'hidden_size': '384', 'epochs': '50', 'batch_size': '32'} | 0.51 | 0.09 | 0.52 | 0.09 |
| baseline | ANN | TRUE | {'learning_rate': '0.001', 'dropout_rate': '0.3', 'hidden_size': '128', 'epochs': '50', 'batch_size': '32'} | 0.51 | 0.06 | 0.52 | 0.05 |
| baseline | ANN | TRUE | {'learning_rate': '0.001', 'dropout_rate': '0.2', 'hidden_size': '64', 'epochs': '50', 'batch_size': '32'} | 0.54 | 0.24 | 0.54 | 0.24 |
| baseline | LogisticRegression | FALSE | {'C': '0.1', 'penalty': 'l2'} | 0.54 | 0.2 | 0.55 | 0.15 |
| baseline | LogisticRegression | FALSE | {'C': '0.01', 'penalty': 'l2'} | 0.54 | 0.25 | 0.54 | 0.25 |
| baseline | LogisticRegression | FALSE | {'C': '0.01', 'penalty': 'l1'} | 0.54 | 0.25 | 0.54 | 0.25 |
| baseline | LogisticRegression | FALSE | {'C': '0.1', 'penalty': 'l1'} | 0.54 | 0.26 | 0.54 | 0.25 |
| baseline | LogisticRegression | TRUE | {'C': '0.1', 'penalty': 'l2'} | 0.55 | 0.09 | 0.55 | 0.08 |
| baseline | LogisticRegression | TRUE | {'C': '0.01', 'penalty': 'l2'} | 0.54 | 0.23 | 0.54 | 0.21 |
| baseline | LogisticRegression | TRUE | {'C': '0.01', 'penalty': 'l1'} | 0.54 | 0.27 | 0.53 | 0.26 |
| baseline | LogisticRegression | TRUE | {'C': '0.1', 'penalty': 'l1'} | 0.54 | 0.13 | 0.54 | 0.11 |
| baseline | XGBoostClassifier | FALSE | {'max_depth': '2', 'learning_rate': '0.001'} | 0.53 | 0 | 0.53 | 0 |
| baseline | XGBoostClassifier | FALSE | {'max_depth': '3', 'learning_rate': '0.01'} | 0.54 | 0.24 | 0.54 | 0.24 |
| baseline | XGBoostClassifier | FALSE | {'max_depth': '2', 'learning_rate': '0.01'} | 0.54 | 0.26 | 0.54 | 0.25 |
| baseline | XGBoostClassifier | TRUE | {'max_depth': '2', 'learning_rate': '0.001'} | 0.5 | 0.32 | 0.51 | 0.29 |
| baseline | XGBoostClassifier | TRUE | {'max_depth': '3', 'learning_rate': '0.01'} | 0.53 | 0.32 | 0.53 | 0.29 |
| baseline | XGBoostClassifier | TRUE | {'max_depth': '2', 'learning_rate': '0.01'} | 0.53 | 0.32 | 0.53 | 0.29 |
| Experiment 1 | ANN | FALSE | {'learning_rate': '0.001', 'dropout_rate': '0.3', 'hidden_size': '384', 'epochs': '50', 'batch_size': '32'} | 0.64 | 0.03 | 0.64 | 0.03 |
| Experiment 1 | ANN | FALSE | {'learning_rate': '0.001', 'dropout_rate': '0.3', 'hidden_size': '128', 'epochs': '50', 'batch_size': '32'} | 0.62 | 0.01 | 0.63 | 0 |
| Experiment 1 | ANN | FALSE | {'learning_rate': '0.001', 'dropout_rate': '0.2', 'hidden_size': '64', 'epochs': '50', 'batch_size': '32'} | 0.61 | 0 | 0.62 | 0 |
| Experiment 1 | ANN | TRUE | {'learning_rate': '0.001', 'dropout_rate': '0.3', 'hidden_size': '384', 'epochs': '50', 'batch_size': '32'} | 0.63 | 0.36 | 0.63 | 0.34 |
| Experiment 1 | ANN | TRUE | {'learning_rate': '0.001', 'dropout_rate': '0.3', 'hidden_size': '128', 'epochs': '50', 'batch_size': '32'} | 0.64 | 0.36 | 0.64 | 0.34 |
| Experiment 1 | ANN | TRUE | {'learning_rate': '0.001', 'dropout_rate': '0.2', 'hidden_size': '64', 'epochs': '50', 'batch_size': '32'} | 0.64 | 0.37 | 0.64 | 0.34 |
| Experiment 1 | LogisticRegression | FALSE | {'C': '0.1', 'penalty': 'l2'} | 0.64 | 0.36 | 0.64 | 0.34 |
| Experiment 1 | LogisticRegression | FALSE | {'C': '0.01', 'penalty': 'l2'} | 0.64 | 0.37 | 0.64 | 0.34 |
| Experiment 1 | LogisticRegression | FALSE | {'C': '0.01', 'penalty': 'l1'} | 0.62 | 0.35 | 0.62 | 0.33 |
| Experiment 1 | LogisticRegression | FALSE | {'C': '0.1', 'penalty': 'l1'} | 0.64 | 0.37 | 0.64 | 0.35 |
| Experiment 1 | LogisticRegression | TRUE | {'C': '0.1', 'penalty': 'l2'} | 0.64 | 0.36 | 0.64 | 0.34 |
| Experiment 1 | LogisticRegression | TRUE | {'C': '0.01', 'penalty': 'l2'} | 0.64 | 0.37 | 0.64 | 0.34 |
| Experiment 1 | LogisticRegression | TRUE | {'C': '0.01', 'penalty': 'l1'} | 0.63 | 0.35 | 0.63 | 0.33 |
| Experiment 1 | LogisticRegression | TRUE | {'C': '0.1', 'penalty': 'l1'} | 0.64 | 0.37 | 0.64 | 0.34 |
| Experiment 1 | XGBoostClassifier | FALSE | {'max_depth': '2', 'learning_rate': '0.001'} | 0.64 | 0 | 0.63 | 0 |
| Experiment 1 | XGBoostClassifier | FALSE | {'max_depth': '3', 'learning_rate': '0.01'} | 0.65 | 0.38 | 0.65 | 0.33 |
| Experiment 1 | XGBoostClassifier | FALSE | {'max_depth': '2', 'learning_rate': '0.01'} | 0.65 | 0.38 | 0.64 | 0.34 |
| Experiment 1 | XGBoostClassifier | TRUE | {'max_depth': '2', 'learning_rate': '0.001'} | 0.6 | 0.32 | 0.6 | 0.29 |
| Experiment 1 | XGBoostClassifier | TRUE | {'max_depth': '3', 'learning_rate': '0.01'} | 0.64 | 0.32 | 0.65 | 0.29 |
| Experiment 1 | XGBoostClassifier | TRUE | {'max_depth': '2', 'learning_rate': '0.01'} | 0.64 | 0.32 | 0.64 | 0.29 |
| Experiment 2 | ANN | FALSE | {'learning_rate': '0.001', 'dropout_rate': '0.3', 'hidden_size': '384', 'epochs': '50', 'batch_size': '32'} | 0.62 | 0.04 | 0.61 | 0.03 |
| Experiment 2 | ANN | FALSE | {'learning_rate': '0.001', 'dropout_rate': '0.3', 'hidden_size': '128', 'epochs': '50', 'batch_size': '32'} | 0.61 | 0.02 | 0.6 | 0.02 |
| Experiment 2 | ANN | FALSE | {'learning_rate': '0.001', 'dropout_rate': '0.2', 'hidden_size': '64', 'epochs': '50', 'batch_size': '32'} | 0.59 | 0 | 0.59 | 0 |
| Experiment 2 | ANN | TRUE | {'learning_rate': '0.001', 'dropout_rate': '0.3', 'hidden_size': '384', 'epochs': '50', 'batch_size': '32'} | 0.6 | 0.33 | 0.61 | 0.32 |
| Experiment 2 | ANN | TRUE | {'learning_rate': '0.001', 'dropout_rate': '0.3', 'hidden_size': '128', 'epochs': '50', 'batch_size': '32'} | 0.6 | 0.34 | 0.61 | 0.32 |
| Experiment 2 | ANN | TRUE | {'learning_rate': '0.001', 'dropout_rate': '0.2', 'hidden_size': '64', 'epochs': '50', 'batch_size': '32'} | 0.6 | 0.34 | 0.61 | 0.32 |
| Experiment 2 | LogisticRegression | FALSE | {'C': '0.1', 'penalty': 'l2'} | 0.62 | 0.35 | 0.62 | 0.32 |
| Experiment 2 | LogisticRegression | FALSE | {'C': '0.01', 'penalty': 'l2'} | 0.62 | 0.37 | 0.62 | 0.33 |
| Experiment 2 | LogisticRegression | FALSE | {'C': '0.01', 'penalty': 'l1'} | 0.6 | 0.32 | 0.59 | 0.31 |
| Experiment 2 | LogisticRegression | FALSE | {'C': '0.1', 'penalty': 'l1'} | 0.63 | 0.35 | 0.62 | 0.32 |
| Experiment 2 | LogisticRegression | TRUE | {'C': '0.1', 'penalty': 'l2'} | 0.6 | 0.34 | 0.61 | 0.32 |
| Experiment 2 | LogisticRegression | TRUE | {'C': '0.01', 'penalty': 'l2'} | 0.61 | 0.34 | 0.61 | 0.32 |
| Experiment 2 | LogisticRegression | TRUE | {'C': '0.01', 'penalty': 'l1'} | 0.6 | 0.33 | 0.59 | 0.31 |
| Experiment 2 | LogisticRegression | TRUE | {'C': '0.1', 'penalty': 'l1'} | 0.61 | 0.35 | 0.61 | 0.32 |
| Experiment 2 | XGBoostClassifier | FALSE | {'max_depth': '2', 'learning_rate': '0.001'} | 0.62 | 0 | 0.63 | 0 |
| Experiment 2 | XGBoostClassifier | FALSE | {'max_depth': '3', 'learning_rate': '0.01'} | 0.63 | 0.36 | 0.65 | 0.35 |
| Experiment 2 | XGBoostClassifier | FALSE | {'max_depth': '2', 'learning_rate': '0.01'} | 0.63 | 0.36 | 0.64 | 0.34 |
| Experiment 2 | XGBoostClassifier | TRUE | {'max_depth': '2', 'learning_rate': '0.001'} | 0.59 | 0.32 | 0.6 | 0.29 |
| Experiment 2 | XGBoostClassifier | TRUE | {'max_depth': '3', 'learning_rate': '0.01'} | 0.62 | 0.32 | 0.62 | 0.29 |
| Experiment 2 | XGBoostClassifier | TRUE | {'max_depth': '2', 'learning_rate': '0.01'} | 0.62 | 0.32 | 0.62 | 0.29 |
| Experiment 3 | ANN | FALSE | {'learning_rate': '0.001', 'dropout_rate': '0.3', 'hidden_size': '384', 'epochs': '50', 'batch_size': '32'} | 0.6 | 0.03 | 0.59 | 0.03 |
| Experiment 3 | ANN | FALSE | {'learning_rate': '0.001', 'dropout_rate': '0.3', 'hidden_size': '128', 'epochs': '50', 'batch_size': '32'} | 0.54 | 0 | 0.53 | 0 |
| Experiment 3 | ANN | FALSE | {'learning_rate': '0.001', 'dropout_rate': '0.2', 'hidden_size': '64', 'epochs': '50', 'batch_size': '32'} | 0.48 | 0 | 0.47 | 0.01 |
| Experiment 3 | ANN | TRUE | {'learning_rate': '0.001', 'dropout_rate': '0.3', 'hidden_size': '384', 'epochs': '50', 'batch_size': '32'} | 0.59 | 0.33 | 0.59 | 0.31 |
| Experiment 3 | ANN | TRUE | {'learning_rate': '0.001', 'dropout_rate': '0.3', 'hidden_size': '128', 'epochs': '50', 'batch_size': '32'} | 0.57 | 0.3 | 0.56 | 0.26 |
| Experiment 3 | ANN | TRUE | {'learning_rate': '0.001', 'dropout_rate': '0.2', 'hidden_size': '64', 'epochs': '50', 'batch_size': '32'} | 0.58 | 0.31 | 0.58 | 0.29 |
| Experiment 3 | LogisticRegression | FALSE | {'C': '0.1', 'penalty': 'l2'} | 0.59 | 0.32 | 0.57 | 0.29 |
| Experiment 3 | LogisticRegression | FALSE | {'C': '0.01', 'penalty': 'l2'} | 0.59 | 0.33 | 0.59 | 0.3 |
| Experiment 3 | LogisticRegression | FALSE | {'C': '0.01', 'penalty': 'l1'} | 0.58 | 0.32 | 0.59 | 0.3 |
| Experiment 3 | LogisticRegression | FALSE | {'C': '0.1', 'penalty': 'l1'} | 0.59 | 0.32 | 0.59 | 0.3 |
| Experiment 3 | LogisticRegression | TRUE | {'C': '0.1', 'penalty': 'l2'} | 0.57 | 0.31 | 0.57 | 0.29 |
| Experiment 3 | LogisticRegression | TRUE | {'C': '0.01', 'penalty': 'l2'} | 0.59 | 0.33 | 0.58 | 0.29 |
| Experiment 3 | LogisticRegression | TRUE | {'C': '0.01', 'penalty': 'l1'} | 0.58 | 0.32 | 0.59 | 0.3 |
| Experiment 3 | LogisticRegression | TRUE | {'C': '0.1', 'penalty': 'l1'} | 0.59 | 0.33 | 0.59 | 0.29 |
| Experiment 3 | XGBoostClassifier | FALSE | {'max_depth': '2', 'learning_rate': '0.001'} | 0.58 | 0.1 | 0.57 | 0.11 |
| Experiment 3 | XGBoostClassifier | FALSE | {'max_depth': '3', 'learning_rate': '0.01'} | 0.6 | 0.31 | 0.59 | 0.28 |
| Experiment 3 | XGBoostClassifier | FALSE | {'max_depth': '2', 'learning_rate': '0.01'} | 0.58 | 0.32 | 0.58 | 0.29 |
| Experiment 3 | XGBoostClassifier | TRUE | {'max_depth': '2', 'learning_rate': '0.001'} | 0.56 | 0.32 | 0.57 | 0.29 |
| Experiment 3 | XGBoostClassifier | TRUE | {'max_depth': '3', 'learning_rate': '0.01'} | 0.58 | 0.32 | 0.56 | 0.29 |
| Experiment 3 | XGBoostClassifier | TRUE | {'max_depth': '2', 'learning_rate': '0.01'} | 0.58 | 0.32 | 0.58 | 0.29 |
